# The time course of emotional picture processing: an event-related potential study using a rapid serial visual presentation paradigm

**DOI:** 10.3389/fpsyg.2015.00954

**Published:** 2015-07-09

**Authors:** Chuanlin Zhu, Weiqi He, Zhengyang Qi, Lili Wang, Dongqing Song, Lei Zhan, Shengnan Yi, Yuejia Luo, Wenbo Luo

**Affiliations:** ^1^Research Center of Brain and Cognitive Neuroscience, School of Psychology, Liaoning Normal University, DalianChina; ^2^School of Educational Science, Huaiyin Normal University, HuaiyinChina; ^3^School of Education Science, Liaocheng University, LiaochengChina; ^4^Institute of Affective and Social Neuroscience, Shenzhen University, ShenzhenChina; ^5^Laboratory of Cognition and Mental Health, Chongqing University of Arts and Sciences, ChongqingChina

**Keywords:** emotional pictures, event-related potentials, rapid serial visual presentation, P2, late positive potential

## Abstract

The present study recorded event-related potentials using rapid serial visual presentation paradigm to explore the time course of emotionally charged pictures. Participants completed a dual-target task as quickly and accurately as possible, in which they were asked to judge the gender of the person depicted (task 1) and the valence (positive, neutral, or negative) of the given picture (task 2). The results showed that the amplitudes of the P2 component were larger for emotional pictures than they were for neutral pictures, and this finding represents brain processes that distinguish emotional stimuli from non-emotional stimuli. Furthermore, positive, neutral, and negative pictures elicited late positive potentials with different amplitudes, implying that the differences between emotions are recognized. Additionally, the time course for emotional picture processing was consistent with the latter two stages of a three-stage model derived from studies on emotional facial expression processing and emotional adjective processing. The results of the present study indicate that in the three-stage model of emotion processing, the middle and late stages are more universal and stable, and thus occur at similar time points when using different stimuli (faces, words, or scenes).

## Introduction

Rapid responses to emotional arousal stimuli, especially potentially biologically relevant stimuli, such as snakes, tigers, or pictures of accidents, particularly when attentional resources are limited, is believed to be evolutionarily significant to humans (Schupp et al., 2006; Vuilleumier and Pourtois, 2007). Understanding the temporal characteristics of rapid emotion processing can help improve emotion recognition, allowing us to make the proper response. In fact, many studies have explored the time course of emotion processing; in these studies, different stimuli, such as emotional words, facial expressions, and sounds, have been employed to induce different emotions. In addition, given its high temporal resolution, the event-related potential (ERP) technique has been widely adopted in studies of emotion processing.

Recently, researchers have begun to investigate the timing of emotional facial expressions processing. For example, the results of an ERP study by Utama et al. (2009), which used emotional facial images as stimuli and required the participants to distinguish the type of emotion and rate the emotional intensity of each image, revealed that the P1 component was significantly correlated with the correct recognition of facial images, while the N170 was significantly correlated with the intensity level rating of the images. Thus, the authors concluded that processing facial emotion is comprised of two different stages: (1) the rapid recognition of facial emotions, which occurs as early as 100 ms after image onset, and (2) detailed processing, such as the intensity assessment, which occurs around 170 ms post-stimulus.

Though the above mentioned two-stage model of emotional facial expression processing revealed the time course of emotional facial processing to some extent, it seems too rough to clarify the temporal characteristics 100 ms after image presentation. To solve this problem, based on the rapid serial visual presentation (RSVP) paradigm, Luo et al. (2010), who adopted emotional facial pictures (from the Chinese Facial Affective Picture System, CFAPS) as stimuli, proposed a three-stage model of facial expression processing? In this model, the brain distinguishes negative emotional facial expressions from positive and neutral facial expressions in the first stage, which explains why the posterior P1 and anterior N1 amplitudes elicited by fearful faces are larger than the amplitudes elicited by neutral and happy faces. In the second stage, the brain distinguishes emotional from non-emotional facial expressions, which explains why the N170 and vertex positive potential (VPP) amplitudes elicited by fearful and happy faces are larger than those elicited by neutral faces. In the third stage, the brain classifies different types of facial expressions, thus explaining why the P3 and N3 amplitudes elicited by fearful, happy, and neutral faces are different from one another. This three-stage model may help us to understand the time courses of emotional facial expression processing. Below, we introduce several representative ERP components that are involved in emotion processing.

When it comes to early emotional facial expressions processing, we cannot ignore two representative ERP components, namely P1 and N1. Both of these components are indicators of early processing (Brown et al., 2012) and represent comparatively automatic mechanisms of selective attention (Dennis et al., 2009). P1 is a positive-going potential that peaks around 80–130 ms after stimulus onset, and is presumed to indicate early visual processing (Jessen and Grossmann, 2014). Furthermore, it reaches its maximal amplitude over the occipital areas in emotional word and facial expression processing (Van Hooff et al., 2008; Cunningham et al., 2012). Moreover, P1 is related to the selective attention to emotional stimuli, i.e., the P1 amplitudes elicited by attended stimuli were higher than the P1 amplitudes to stimuli that were unattended (Dennis et al., 2009). Additionally, the P1 amplitudes elicited by negative emotional pictures and words were larger than the P1 amplitudes elicited by positive ones (Bernat et al., 2001; Smith et al., 2003; Delplanque et al., 2004). N1, a negative-going potential, appears shortly after P1, and is sensitive to the characteristics of facial expressions; for example, Eimer and Holmes (2002) found that fearful faces induced a shorter N1 latency than did neutral faces.

Another important component is P2. P2 is an attention-related component, with a typical peak latency of about 200–250 ms (Ferreira-Santos et al., 2012), which reflects the detection of visual features during the perceptual stage of processing (Luck and Hillyard, 1994; Carretié and Iglesias, 1995). Moreover, P2 is regarded as indexing some aspects of the stimulus categorization process (García-Larrea et al., 1992). Previous ERP studies on the relationship between the valence of stimuli and the P2 amplitude present conflicting results. On one hand, using both emotional pictures (Carretié et al., 2004) and emotional words (Herbert et al., 2006; Kanske and Kotz, 2007) as stimuli, researchers found that emotional stimuli elicited significantly larger P2 amplitudes than did neutral stimuli. However, Yuan et al. (2007) reported that the P2 amplitudes elicited by emotional and neutral stimuli were not significantly different. At the same time, some researchers found that the P2 amplitudes elicited by negative stimuli were significantly greater than those elicited by positive stimuli (Carretié et al., 2001; Delplanque et al., 2004; Huang and Luo, 2006; Wang and Bastiaansen, 2014), while others have reached the opposite conclusion (Schapkin et al., 1999). Thus, the exact role of the P2 component in emotion processing remains unclear.

The late positive potential (LPP, also known as LPC) is a positive, slow components elicited by both emotional (positive and negative) and neutral stimuli, and can be used as an indicator of continued attention to a motivationally striking stimulus (Jaworska et al., 2011; Weinberg and Hajcak, 2011; Leite et al., 2012; Gable and Adams, 2013; Novosel et al., 2014). Numerous studies (Dennis and Hajcak, 2009; Hajcak et al., 2009, 2013; Kessel et al., 2013; Smith et al., 2013; Yuan et al., 2014) showed that the amplitude of the LPP is sensitive to stimulus valence, i.e., emotional stimuli (positive and negative) elicited larger LPP amplitudes than did neutral stimuli. However, whether positive and negative stimuli differentially affect the LPP amplitude has not yet been agreed upon by researchers. By adopting emotional pictures from the International Affective Picture System (IAPS) as stimuli and controlling for the arousal dimension of the pictures, Schupp et al. (2004) and Codispoti et al. (2006) found that the LPP amplitudes elicited by positively and negatively valenced pictures were not significantly different in healthy subjects. In contrast, Huang and Luo (2009) and Weinberg and Hajcak (2010) demonstrated that unpleasant stimuli elicited larger LPP amplitudes than did pleasant stimuli. Hence, more research is necessary to resolve these inconsistencies.

Besides conveying emotional information through facial expressions, humans can convey emotional information through writing and speech, which inspired researchers to explore the time course of emotional word processing. Thus, with emotional words, the findings were quite similar to those obtained with faces, although some differences were also observed. However, our later studies indicate that, in general, the three-stage model still holds. For example, in one of our recent studies (Zhang et al., 2014), we adopted emotional Chinese adjective (selected from the Chinese Affective Word System, CAWS), and used the same paradigm (RSVP) as that in our previous study (Luo et al., 2010). In that study, we found that the processing of emotional adjective seemed to occur in the same three stages as did the processing of emotional facial expressions. Specifically, in the first stage, the brain distinguished negative adjectives from non-negative adjectives. Next, the brain discerned emotional adjectives from non-emotional adjectives. Lastly, negative, neutral, and positive adjective were separated. Soon after, in a separate study, we investigated the time course of emotional noun processing and found that the processing of emotional nouns (from the CAWS) also occurred in three different stages (Yi et al., 2015). Besides, we did not find the negativity bias for emotional noun processing that is usually found in the processing of emotional facial expressions and adjectives, i.e., the brain did not distinguish negative nouns from positive and neutral nouns in the first stage. The remaining two stages of emotional noun processing showed similar results to the results for emotional adjective processing, indicating that the brain distinguished emotional nouns from non-emotional nouns in the middle stage, and classified the three different types of nouns (negative, positive, and neutral) in the last stage. These major findings for the time courses of emotional facial expression and word processing are summarized in **Table 1**.

**Table 1:** Summary of findings.

Stimuli/ERP/Time	First stage	Second stage	Third stage
Facial expression	P1, N1: R > L neg > neu, pos (ns)	N170: R > L VPP: M > R, L neg, pos (ns) > neu	N300: R > M, L P300: M, R > L neg > pos > neu
Emotional adjective	P1:L, R > M Left:neg > neu, pos (ns)	N170, EPN L: neg, pos (ns) > neu R:neg, pos vs. neu (ns)	LPC:M > R, Lvs. R, M (ns) pos > neg > neu
Emotional noun	PI pos, neu vs. neg (ns) pos vs. neu (ns)	N170: L > R L: pos, neg > neu R:pos, neg vs. neu (ns)	LPC:M > R, L vs. R, M (ns) neg > neu > pos

*Comparisons of the time courses of emotional facial expression and word processing based on representative ERP components. neg, negative; neu, neutral; pos, positive; ns, no significant difference; R, L, and M = Right, left, and middle hemisphere.*

The existence of three temporal processing stages for emotional facial expression and word processing is easily demonstrated. However, there are also processing differences between the two stimulus types. Firstly, we found that negative expressions and adjectives elicited lager right (Luo et al., 2010) and left (Zhang et al., 2014) N1 amplitudes, respectively, than did positive and neutral expressions and adjectives; however, the N1 amplitudes elicited by negative, neutral, and positive nouns were not significantly different (Yi et al., 2015). Secondly, although the N170 effect has been found in all processes, the right hemisphere appears to be superior for processing emotional facial expressions, but the left hemisphere appears to be superior for word (both emotional adjective and noun) processing. These discrepancies may be explained by the different types of stimuli (facial expressions versus words) adopted in the two experiments. Indeed, the processing differences between these two common emotion inducing materials [words and pictures (facial expressions)] have been widely discussed in past years. Firstly, formally speaking, words transmit affective information on a symbolic level, while facial expressions convey emotion information through biological cues (Zhang et al., 2014). Comparatively, the latter is more direct, which is relevant to the processing difference between the two stimuli. Next, some researchers (Seifert, 1997; Azizian et al., 2006) discovered that processing superiority exists for pictures vs. words, i.e., pictures have quicker and simpler access to associations. Since words convey symbolic information, it is reasonable to suspect that the processing of words requires extra translation activity at a surface level before they access the semantic system. Additionally, different brain regions have been activated when processing pictures and words. A positron emission tomography (PET) experiment (Grady et al., 1998) has shown greater activity of the bilateral visual pathway and medial temporal cortices, during processing of pictures. In contrast, for processing of words, strong activation patterns was observed in prefrontal and temporoparietal regions.

As mentioned above, it has been showed that the processing of both emotional facial expressions and words occurs in three different stages; however, what about the time course of emotional pictures that are not of faces (e.g., scenes)? Considering the potential similarities between processing emotional facial expressions and pictures (for example, both types of stimuli could transmit emotion information more intuitively than words), we postulated that the processing of emotional pictures would also show different stages. The present study, adopting the RSVP paradigm and using both neutral faces and scenes as stimuli, aimed to explore the temporal characteristics of emotional picture processing under conditions of limited attentional resources. It is reasonable to speculate that the processing of emotional pictures will show similar temporal characteristics to those shown in facial expression processing.

The early ERP components are sensitive to the non-emotional perceptual features of the stimuli (De Cesarei and Codispoti, 2006; Olofsson et al., 2008). It has been found that in the earliest processing stages, the physical properties of a stimulus such as its color (Cano et al., 2009) and complexity (the latter producing a very early effect, i.e., 150 ms after stimulus onset; Olofsson et al., 2008) influence the affective waveforms. Considering these factors, it is reasonable to expect that the processing of emotional pictures in the early stage is not pure processing to isolate the emotion information conveyed by the pictures; therefore, it is not possible to distinguish whether the main effect was caused by physical attributes or emotions, even if there appeared to be a P1 main effect. Based on these considerations, the early processing of emotional pictures was not analyzed in the present study. We hypothesized that subjects would distinguish emotional pictures from non-emotional pictures in the middle stage of emotional picture processing and would distinguish the different valences of the pictures in the late stage.

## Materials and Methods

### Subjects

Eighteen (nine men) healthy undergraduates from Chongqing University of Arts and Sciences were tested. All participants reported normal or corrected to normal visual acuity and no history of mental illness and brain disease, and all of them were right-handed. Participants received a small amount of money for participation. All participants provided written informed consent, which was approved by the Ethics Committee of The Chongqing University of Arts and Sciences.

### Stimuli

Materials consisted of four neutral face pictures, 18 emotional pictures, and 12 scrambled pictures (SPs), described in detail below. Four grayscale photographs of four different identities (two females) showing neutral expressions were selected from the CFAPS, while eighteen emotional pictures convey positive, neutral, or negative emotions (six neutral, six happy, and six fearful pictures) were selected from the Chinese Affective Picture System (CAPS). The emotional pictures were not face-related pictures, including landscapes, animals and events of life scenes. SPs made by randomly swapping small parts (18 × 18 pixels) of the same neutral pictures were used as distraction stimuli. The scrambled images had the same rectangular shape, size, luminance, and spatial frequency as the face pictures and emotional pictures, used as mask. The visual angle was 5.6 × 4.2°. To control the influence of arousal on the processing of emotional pictures, the arousals of all images having been measured on a 9-point scale before the formal experiment, the arousals of emotional pictures (M ± SD, positive: 5.61 ± 0.43, negative: 5.97 ± 0.59) were higher than neutral pictures (M ± SD, 3.76 ± 0.36), *F*(2,15) = 8.40, *p* < 0.001, η^2^ = 0.836, and there was no difference between positive and negative pictures (*p* = 0.607), while the emotional pictures differed significantly in valence, *F*(2,15) = 434.49, *p* < 0.001, η^2^ = 0.983; M ± SD, positive: 7.61 ± 0.15, neutral: 4.81 ± 0.23, negative: 2.53 ± 0.44.

### Procedure

**FIGURE 1:**
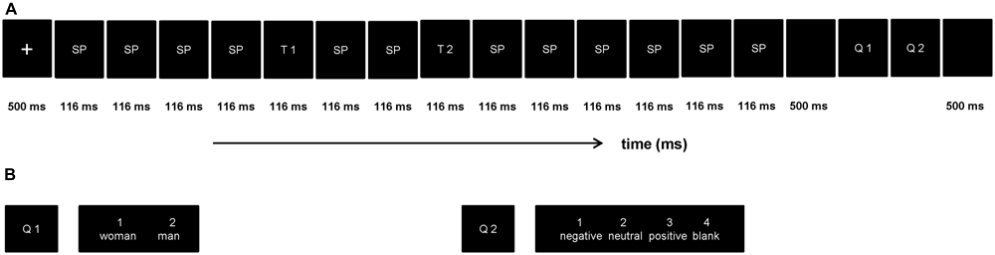
**The rapid serial visual presentation (RSVP) paradigm used in this experiment. a(A)** Each trial contained 10 scrambled pictures (SP), two target stimuli (T1 and T2), and two questions (Q1 and Q2). The T1 emerged randomly and equi-probably at the fifth, sixth, seventh position, T1 and T2 were presented within an interval of 232 ms. **(B)** Example for the two questions in each trail.

In order to restrict attention resources, the procedure of present ERP study was designed on the basis of the RSVP paradigm. The procedure includes four blocks, while each block comprised of 120 trials, a total of 480 trials. As showed in **Figure 1**, each trial structure consisted of fourteen pictures (two target stimulus), pictures were portrayed with a stimulus-onset asynchrony (SOA) of 116 ms and no blank inter stimulus interval (ISI). The T1 emerged randomly and equi-probably at the fifth, sixth, seventh position, nextly, two distracting stimulus, then the T2 (SOA = 348 ms), and the remaining distracting stimulus items were presented in turn succesively. All stimuli were presented in the center of the screen. The first target stimulus (T1, discriminate the gender showed in the picture, subjects were asked to press Key “1” when the stimuli was woman and Key “2” when if it was man) was one of the four gender pictures (two females) and the second target stimulus (T2, discriminate the valence showed in the picture, press Key “1” if T2 was negative, Key “2” when neutral, Key “3” when positive, Key “4” if T2 was blank) was one of the 18 emotion pictures. The question would disappear while participants pressed the index key, and this task without a specific time limit. All subjects were required to respond to the two questions with their right hand. Stimulus presentation was controlled by the E-Prime 1.1 software (Psychology Software Tools Inc., Pittsburgh, PA, USA).

This study was completed in a sound attenuated room, participants sat in front of a 17″ computer screen (refresh rate 60 Hz) at a distance of 90 cm. In order to ensure them fully understand the experiment procedure, with 20 practice trials before the formal experiment. In front of each trial, a white fixation point present in the center of the screen for 500 ms. After the presentation of each trial, participants were asked to complete both task 1 and task 2 as accurately as possible. In terms of time sequence, they were told to complete task 2 as accurately as possible, based on the accurate response to task 1. The two tasks were appeared serially with fixed order at the end of each trial. Then, participants would be led into the following series after 500 ms during which the screen stayed black and blank. No feedback was provided for each trial. To avoid being overtired, all participants were forced to rest for 2 min after each block.

### Data Recording and Analysis

Brain electrical activity was recorded from 64 scalp sites using tin electrodes mounted on an elastic cap (Brain Product, Munich, Germany) referentially against left mastoids. Horizontal electrooculographies (EOGs) were recorded from two electrodes sites at the outer canthi of each eye, while vertical EOGs were recorded from tin electrodes placed 1 cm above and below the right eye. All interelectrode impedances were kept below 5 kΩ. The EOG and electroencephalogram were collected with a bandpass of 0.01–100 Hz, sampled at a rate of 500 Hz, and re-reference oﬄine to obtain a global average (Bentin et al., 1996). The EEG data were corrected for eye movements using the method proposed by Gratton et al. (1983), as implemented in the Brain Vision Analysis software (Version 2.0; Brain Product, Gilching, Germany). Horizontal EOGs and vertical EOGs were used to pick up eye movement artifacts. Trials with EOG artifacts (mean EOG voltage exceeding ±80 μV) and other artifacts (peak-to-peak deflection exceeding ±80 μV) were excluded from averaging. The averages were then digitally filtered (low-cut 30 HZ, 24 dB/octave).

The averaged epoch for ERP was 1200 ms including a 200 ms pre-stimulus baseline. Trials were accepted only if participants gave correct response to both T1 and T2. EEG images evoked by the positive, neutral, and negative emotions were overlaid and averaged. On the basis of the hypothesis and the topographical distribution of grand-averaged ERP activity, P2 and LPP were chosen for statistical analysis in the presented study. The P2 component (150–280 ms) were analyzed at the following nine electrode sites (Fz, F3, F4, FCz, FC3, FC4, Cz, C3, C4), we used local peak detection for each of the nine electrode sites (Fz, F3, F4, FCz, FC3, FC4, Cz, C3, C4) and applied a peak detection over the common (across three conditions) grand average (coming up with nine latencies), which was then commonly used even if a particular participant did not have a peak there, 21 sites (Fz, F3, F4, FCz, FC3, FC4, Cz, C3, C4, CPz, CP3, CP4, Pz, P3, P4, POz, PO3, PO4, Oz, O1, O2) were chosen for the analysis of LPP (400–500 ms). The baseline-to-peak amplitude was computed for P2 component while average amplitude was computed for LPP. Amplitudes and latency of each component were measured by a two-way repeated-measure analysis of variances (ANOVAs). Factors involved in the analysis were emotional types (three levels: positive, neutral and negative) and electrodes site, *p*-values were corrected by Greenhouse-Geisser correction.

## Results

### Behavior Results

The results of ANOVA for the accuracy revealed a significant main effect of emotional type, *F*(2,34) = 5.40, *p* = 0.018, η^2^ = 0.241. The results of pairwise comparison showed that the accuracies of negative emotion pictures (97.06 ± 2.86%) were higher than positive emotion pictures (91.89 ± 7.05%) and neutral pictures (89.56 ± 11.55%), and there was no significant difference between positive and neutral pictures (*p* > 0.05).

### ERP Data Analysis

#### P2

**FIGURE 2:**
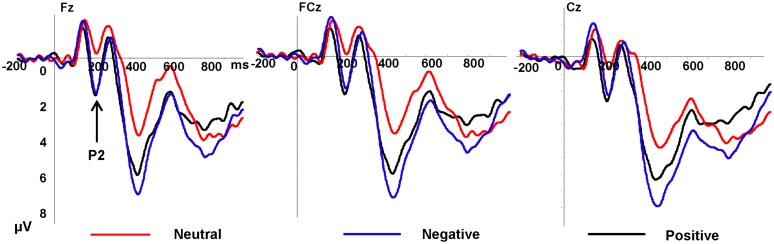
**Grand average event-related potentials (ERPs) of P2 components for negative (purple lines), neutral (red lines), and positive (black lines) conditions recorded at Fz, FCz, Cz**.

As showed in **Figure 2**, the P2 amplitudes revealed significant main effects of emotion type, *F*(2,34) = 8.83, *p* = 0.003, η^2^ = 0.525, and electrode, *F*(8,136) = 3.13, *p* = 0.026, η^2^ = 0.155, there was no significant interaction between emotional type and electrode. Positive pictures (3.29 μV, *p* = 0.001) and negative pictures (3.02 μV, *p* = 0.001) elicited greater amplitudes than neutral pictures (1.27 μV), and the amplitudes elicited by positive and negative pictures showed no significant difference (*p* = 0.562). Frontal electrode elicited lager P2 amplitudes than the other part. The P2 latency showed no significant difference both of main effect and interaction effect.

#### Late Positive Potential

**FIGURE 3:**
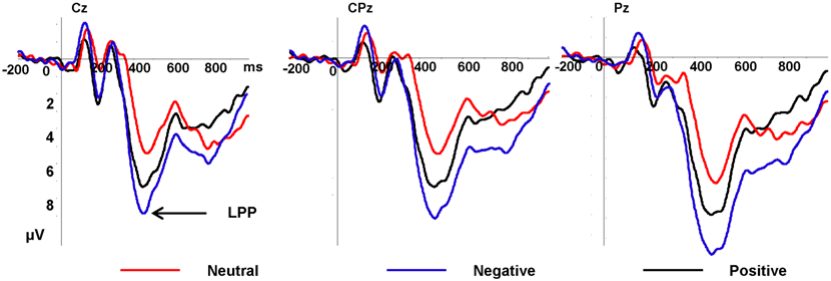
**Grand average ERPs of late positive potential (LPP) components for negative (purple lines), neutral (red lines), and positive (black lines) conditions recorded at Cz, CPz, Pz**.

**FIGURE 4:**
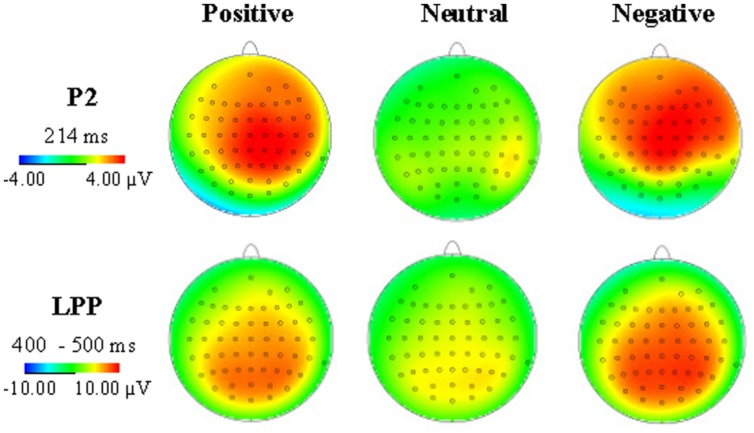
**Average ERP topographies of the P2 and LPP components across three emotional conditions**.

In **Figure 3**, the LPP amplitudes revealed significant main effects of emotion type and electrode [*F*(2,34) = 13.10, *p* < 0.001, η^2^ = 0.435; *F*(20,340) = 4.93, *p* = 0.01], there was no significant interaction between emotional type and electrode. Negative pictures elicited greater (7.69 μV) amplitudes than positive pictures (6.34 μV, *p* = 0.015) and neutral pictures (4.37 μV, *p* < 0.001), and the LPP amplitudes elicited by positive pictures was greater than neutral pictures (*p* = 0.015). Pz electrode elicited the largest LPP amplitudes (7.29 μV, see **Figure 4**).

.

## Discussion

In this study, the behavioral data showed that the participants’ accuracy on negative pictures was higher than their accuracies on positive and neutral pictures (showed no difference). This is in line with the notion that negative stimuli are prioritized in emotion processing given their strong biological significance. Furthermore, the ERP results showed that positive and negative pictures (no difference) elicited larger P2 amplitudes than did neutral pictures. Additionally, the LPP amplitudes elicited by negative pictures were larger than those elicited by non-negative pictures, while positive pictures elicited larger LPP amplitudes than did neutral ones.

In the RSVP paradigm, a series of stimuli, such as pictures, letters, words, or digits is presented in rapid succession at the same location. Usually, the stimuli are presented at the rate of 6–20 item per second, and the participants are instructed to only react to one or more stimuli (the target stimuli) that are different from the other stimuli in the series (distraction stimuli) in color, brightness or other features (Broadbent and Broadbent, 1987; Raymond et al., 1992; Isaak et al., 1999). Many studies (Broadbent and Broadbent, 1987; Weichselgartner and Sperling, 1987; Raymond et al., 1992; Isaak et al., 1999) have indicated that this special paradigm is useful for exploring the temporal characteristics of attentive processes. Additionally, previous studies (Chun, 1997; Kessler et al., 2005; Shapiro et al., 2006) revealed that the attentional resources and task difficulty were affected by two factors, the inter-stimulus interval (the attentional blink appeared when the inter-stimulus interval ranged from 200 to 500 ms) and the number of tasks (participants perform better on a single-task than on a dual-task, which is referred to as the attentional blink), especially in the middle and late stage of emotion processing (for details, please refer to our previous work). The three-stage model of emotional facial expressions and words processing proposed in our previous studies (Luo et al., 2010; Zhang et al., 2014; Yi et al., 2015), was based on the lack of attentional resources, i.e., a process is in accordance with the three-stage model only when the processing of emotional facial expressions and words is within the attentional blink time window. The present study aimed to test whether the processing of emotional pictures other than faces is also in line, or partly in line, with the three-stage model; therefore, the test needed to be conducted under conditions where the participants lacked attentional resources. Based on the above considerations, the present study adopted the dual-task experimental procedure based on the RSVP paradigm.

Utilizing this paradigm, we found that the P2 amplitude elicited by emotional pictures was higher over the frontal lobes compared to those elicited by neutral pictures. Since P2 is generally viewed as an index of certain stimulus attributes (García-Larrea et al., 1992), the P2 component mainly differentiated emotional stimuli from non-emotional stimuli. Increased P2 amplitudes for emotional pictures compared to neutral pictures have also been found by other researchers (Peng et al., 2012; Wu et al., 2013). In addition, neither the main effect nor the interaction with the P2 latency was significant in the present study, which is supported by the results of Yuan et al. (2007). However, Carretié et al. (2001) stated that shorter P2 latencies were elicited in response to negative pictures than in response to positive pictures. However, there are several differences between their study and the present study. First, a different response method was used, i.e., oral report (Carretié et al., 2001) vs. keyboard press (present study). Second, different stimulus presentation times were used (longer in Carretié et al., 2001); the longer times in the study by Carretié et al. (2001) may have absorbed more attentional resources. The largest P2 amplitudes in the present study were identified at frontal sites at approximately the same position as the maximal VPP amplitudes in our previous work. Additionally, no significant differences were found regarding the interaction between the factors of electrode and emotion. In our previous study (Luo et al., 2010), we found that the brain ERPs were able to distinguish emotional expressions (fearful and happy) from neutral facial expressions, but could not distinguish fearful and happy expressions, during the second stage of emotional facial expression processing, as indicated by larger anterior VPP amplitudes in response to fearful and happy faces than in response to neutral faces. Since the VPP effect is largely specific for facial expression processing, we did not recognize a VPP effect in the present study, instead, we recognized P2 effect, but temporally speaking, the P2 effect corresponded to the VPP effect.

In our previous studies (Luo et al., 2010; Zhang et al., 2013a,b, 2014), we found that the brain could differentiate between three types of emotional stimuli during the third stage of emotion processing. In the present study, as expected, the main effect of emotion on the LPP amplitude was significant; specifically, the LPP amplitude was larger to negatively valenced pictures than it was to positively valenced and neutral pictures, and positively valenced pictures elicited larger LPP amplitudes than did neutral pictures, which was coherent with our previous results. Since both the P3 and LPP (or LPC) belong to the P3 family (González-Villar et al., 2014; Grzybowski et al., 2014), it is reasonable to compare these two components directly. Although researchers (Foti and Hajcak, 2008; Hajcak et al., 2010; Lang and Bradley, 2010; Yen et al., 2010; Zhang et al., 2012b) agree that emotional stimuli elicit larger LPP (P3) amplitudes than do neutral stimuli, they do not agree on the relationship between the LPP amplitudes and the valence of emotional stimuli. Some researchers (Ito et al., 1998; Huang and Luo, 2006; Frühholz et al., 2009) found that the LPP amplitudes elicited by negative stimuli were larger than the LPP amplitudes elicited by positive stimuli, which is in agreement with our results. However, in contrast to the present study, Solomon et al. (2012) found that girls displayed larger LPP amplitudes to neutral pictures than to positive pictures. This difference may be interpreted as the influence of developing cognitive and affective factors related to emotion processing, since Solomon enlisted children (between 5 and 6 years old) as participants, while all of the subjects in the present study were college students. Additionally, using emotional pictures as stimuli (selected from the IAPS), Brown et al. (2013) found that the LPP amplitudes elicited by low-arousal unpleasant, neutral, and low-arousal pleasant pictures were not significantly different from each other, which is in line with our results to some degree. However, the LPP amplitudes elicited by high-arousal unpleasant pictures were larger than those elicited by high-arousal pleasant and neutral pictures (the negativity bias). The differences can be interpreted in terms of different arousal levels, since the LPP is sensitive to the arousal levels of emotional stimuli (McConnell and Shore, 2011; Zhang et al., 2012a). The main effect of electrode was significant, with the LPP amplitude being the largest at the Pz electrode, which is supported by many previous studies (Amrhein et al., 2004; Schupp et al., 2004; Schutter et al., 2004; Smith et al., 2013).

As for the three-stage model, preliminary analysis of a threat during the early stage of processing can help individuals quickly escape from the threat, and therefore has strong adaptive significance (Vuilleumier, 2005; Corbetta et al., 2008; Bertini et al., 2013), but this analysis, although fast, is also very coarse. Emotion processing is a dynamic process; the first stage of the three-stage model of emotional facial processing identifies the negative stimuli, while the second stage distinguishes positive stimuli from neutral stimuli. Thus, if we link the first and second stages, it is easy to state that in the first two stages, the identification of these three emotions is basically completed. This raises the question of whether the third stage is necessary. Our previous experimental results (Luo et al., 2010; Zhang et al., 2014; Yi et al., 2015) suggested that fine processing of the three different emotions occurs; hence, the third stage is complementary to the first and second stages. Therefore, we believe the three-stage model of facial emotional expression processing is more appropriate than the two-stage model (Utama et al., 2009).

Since the arousal level affects the processing of visual stimuli (McConnell and Shore, 2011; Zhang et al., 2012a; Prehn et al., 2014), we originally intended to control for the arousal level when selecting the experimental stimuli. Unfortunately, we found that it was almost impossible to absolutely match the arousal level of neutral and emotional (positive and negative) pictures in our picture system. However, because the arousal level of neutral pictures was lower than the arousal level of positive and negative pictures, this seems to indicate higher ecological validity, since valence and arousal were plotted as horizontal and vertical coordinates, respectively, when establishing axes for evaluating the emotional pictures. The resulting scatter plot formed a slight U-shape. Therefore, the P2 amplitudes elicited by emotional pictures were larger than those elicited by neutral pictures in the present study, which might be due to valence or arousal difference between the stimuli, or to the interaction between valence and arousal; however, we were unable to determine the exact cause given the particularity of the emotional scene. Moreover, the interaction between valence and arousal is a very interesting question that we hope to investigate in future studies.

The present experiment only differs from our previous work Luo et al. (2010) in terms of the experimental stimuli, i.e., emotional scene pictures vs. emotional face pictures, respectively, while all other details remain the same (please refer to our previous work). The results of the present study suggest that, temporally speaking, different processing stages exist within the brain for emotional pictures. We found that the middle and late stages for emotional scene processing correspond to the middle and late stages of the three-stage model of facial expression processing. Because of the inherent limitation caused by the use of different types of stimuli, we could not compare emotion processing in the early phase between studies. The details and information in the emotional pictures varied, and these differences (i.e., physical complexity) could not be controlled for in the present study. Therefore, these factors might have affected the early processing stage. Collectively, the present study confirmed the latter two stages of the three-stage model of facial expression processing exist when processing images other than faces when attentional resources are limited, thus, we provide new evidence in supporting of the hypothesis of different temporal processing stages for emotional visual stimuli. Moreover, the results of the present study indicate that the time course of emotional processing is similar even when the emotions are conveyed by different types of stimuli.

## Conclusion

In the present study, by adopting an RSVP paradigm that limited the participants’ attentional resources, we explored the time course of emotional picture processing. The results demonstrated that emotional picture processing showed different stages (P2, LPP) similar to those observed during the processing of emotional faces. This suggests that in the late perceptual and cognitive processing stages, the time courses for processing the same emotions, regardless of whether they are conveyed by facial expressions, words, and pictures (scenes) are similar.

## Conflict of Interest Statement

The authors declare that the research was conducted in the absence of any commercial or financial relationships that could be construed as a potential conflict of interest.
